# Combined effects of sepsis and extracorporeal membrane oxygenation on left ventricular performance in a murine model

**DOI:** 10.1038/s41598-022-26145-7

**Published:** 2022-12-23

**Authors:** Mukhammad Kayumov, Dowan Kim, Sainath Raman, Graeme MacLaren, In Seok Jeong, Hwa Jin Cho

**Affiliations:** 1 Chonnam National University Graduate School, Gwangju, Republic of Korea; 2 Department of Thoracic and Cardiovascular Surgery, Chonnam National University Hospital and Medical School, 42 Jebong-ro Donggu, Gwangju, Republic of Korea; 3 Centre for Children’s Health Research, The University of Queensland, Brisbane, QLD Australia; 4 Cardiothoracic ICU, National University Hospital, Singapore, Singapore; 5 Department of Pediatrics, Chonnam National University Children’s Hospital and Medical School, 42 Jebong-ro Donggu, Gwangju, Republic of Korea

**Keywords:** Preclinical research, Medical research, Experimental models of disease

## Abstract

Extracorporeal membrane oxygenation (ECMO) may be a viable salvage therapy in selected patients with septic shock. As ECMO use increases, we studied left ventricular (LV) performance during sepsis with and without ECMO using a pressure–volume (PV) loop in a murine model and aimed to understand LV hemodynamics in septic shock with ECMO. The rats were divided into Group 1 (ECMO applied to healthy rats), Group 2 (ECMO for septic rats), Group 3 (Controls, n = 20) and Group 4 (Sepsis induction only, n = 20). The cardiac parameters include end-diastolic volume (EDV), end-systolic volume (ESV), end-diastolic pressure (EDP), and end-systolic pressure (ESP), ejection fraction (EF), end-systolic elastance (Ees), diastolic time constant (Tau) index, arterial elastance (Ea), pressure–volume area (PVA), stroke work (SW), and potential energy (PE). We compared the changes of parameters in all groups. A total of 74 rats were included in the analyses. After 2 h on ECMO, Group 2 was associated with significant increases in ESP, EDV, ESV, PVA, PE, and SW. The difference ratio of PE and PVA was significantly higher in Group 2 compared to Group 1 (P < 0.01). In conclusion, myocardial oxygen consumption was higher in septic shock with ECMO than in controls.

## Introduction

Septic shock can lead to life-threatening organ failure due to a dysregulated host response against infection^1,2^. Despite advanced supportive critical care and targeted antimicrobial therapies, mortality remains high^3^. Although there is increasing interest in using extracorporeal membrane oxygenation (ECMO) as rescue therapy in septic shock unresponsive to conventional treatment^4–7^, the role of ECMO for septic shock patients is still under debate^8–12^. As ECMO use increases, there is a need to better understand the cardiovascular effects of ECMO in the context of septic shock.

Cardiovascular features of septic shock are tachycardia, alteration in vascular tone and permeability (with ensuing hypotension), and reduced ventricular function. Heart failure with refractory shock is one of the most severe manifestations of Sepsis^13^. ECMO may be a viable salvage therapy for those with severe sepsis-induced myocardial depression^7^. The hemodynamic effect of ECMO in septic shock also needs to be investigated. The mechanistic rationale for the benefit of ECMO in septic shock is that ECMO can reverse impaired tissue oxygen delivery and myocardial depression by restoring perfusion pressure and increasing systemic oxygen delivery^13,14^. While left ventricular (LV) hemodynamics can be assessed using pressure–volume (PV) loops, detailed LV function during ECMO support for septic shock is yet to be elucidated.

We studied the left ventricular performance during ECMO with and without Sepsis using PV loop measurements in murine models. This study aims to understand LV hemodynamics and elucidate the complex interactions between the heart, vasculature, and ECMO with sepsis.

## Materials and methods

### Animal preparation

This study included forty (n = 20 in each group) male Sprague–Dawley rats (Samtako Bio Korea Co. Ltd., Osan city, Korea) weighing 450–550 g. All rats received human-quality care in compliance with the principles of laboratory animal care formulated by the National Society for Animal Research for the Care and Use of Laboratory Animal Resources. The Chonnam National University Medical School Ethics Committee approved the study (approval No.: CNUH-IACUC-20017) and the study is reported in accordance with ARRIVE guidelines (https://arriveguidelines.org). All animals were anesthetized via intramuscular injection of ketamine (80 mg/kg) and xylazine (8 mg/kg). Isoflurane inhalation was maintained during all surgical procedures. Each rat was intubated and mechanically ventilated with 90% oxygen and 1–1.5% isoflurane via a rodent respirator (Harvard Apparatus Inc.; Holliston, MA, USA). The tidal volume was 8 mL/kg, and the respiratory rate was 55 breaths/min. All experimental rats were euthanized by CO_2_ asphyxiation at the end of the experiments.

### Model establishment: sepsis and ECMO (Fig. 1)

**Figure 1:**
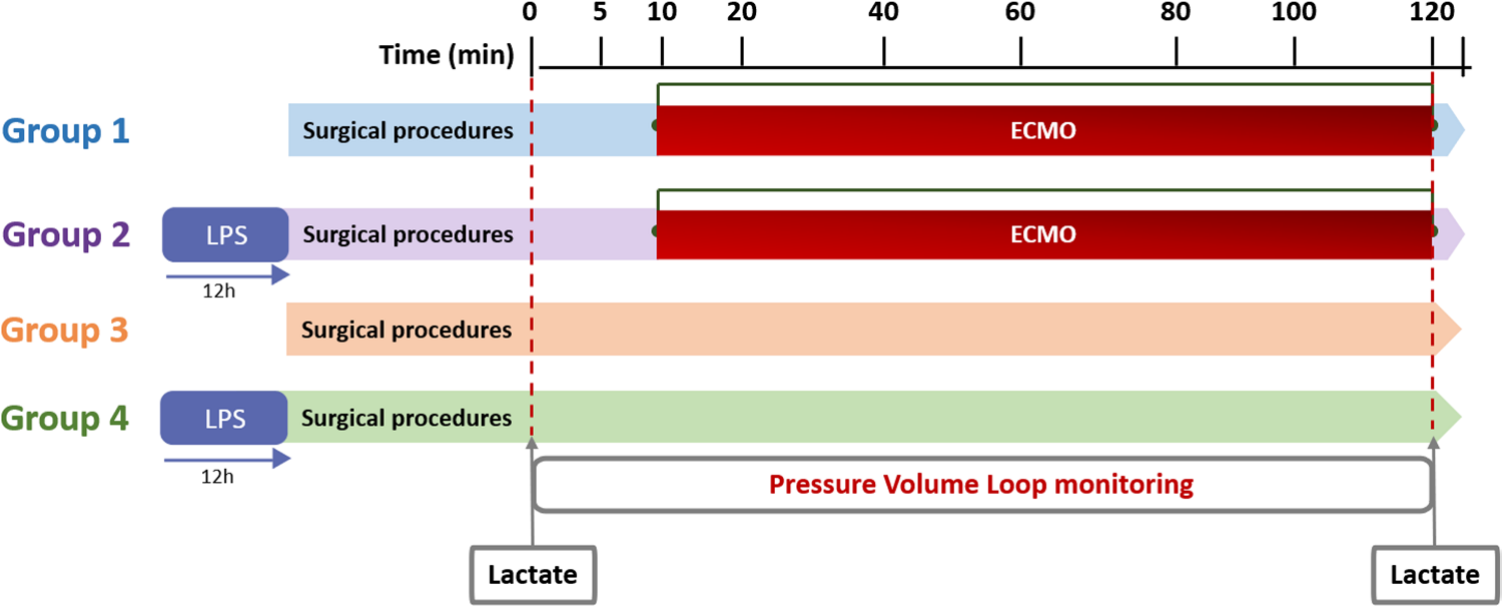
Experimental timetable in each group. Group 1 represents healthy rats with ECMO application. In this group, the PV loop was monitored during the ECMO application. Group 2 represents rats with LPS injection and ECMO application after 12 h. In this group, the PV loop was monitored during the ECMO application. Group 3 represents healthy controls with PV loop monitoring and Group 4 represents septic rat controls with PV loop monitoring.

The rats were classified into Group 1 (ECMO applied to healthy rats, n = 20), Group 2 (ECMO for septic rats, n = 20), Group 3 (the healthy controls, n = 20) and Group 4 (the Sepsis controls, n = 20). Sepsis was induced according to a previously validated method^15^ by injecting 10 mg/kg lipopolysaccharide (LPS, Escherichia coli 0111:B4 diluted in PBS solution 100:1, Sigma-Aldrich, Korea) intraperitoneally 12 h before the start of the experiment. Blood pressure (BP) was monitored after 12 h of LPS injection.

The ECMO circuit configuration employed has been described previously^16^. ECMO cannulation was done via the right jugular vein and left femoral artery. A modified neonatal feeding tube was used for the drainage cannula, and a 24 G angiocatheter was used as the arterial return cannula^16^.

A 2 Fr. Millar catheter (Model: SPR-838. Millar Inc. 6001-A Gulf Freeway, Houston, TX, 77023-5417, USA) was placed into the left ventricle through the right common carotid artery to obtain cardiovascular parameters by PV loop recording. For BP monitoring and blood sampling, the contralateral femoral artery was cannulated with a 24 G angiocatheter.

The Flowmeter sensor (ME3PXL-M5, Transonic Systems Inc. 34 Dutch Mill Rd, Ithaca, New York, 14850, USA) was placed at the drainage site of the ECMO circuit, and ECMO flow was monitored with the corresponding device ("Transonic" tubing flow module, Transonic Systems Inc., Ithaca, NY, 14850, USA). ECMO flow was continuously monitored during the time of ECMO run.

Lactate level was performed using the GEM Premier 3000 system (Werfen; Bedford, MA, USA) employing disposable cartridges. Lactate was measured in the beginning of the experiment and at the end of the experiment. Endotoxin assay analysis were obtained by VERSA max microplate reader and WinKOCLTMsoftware (molecular devices, LLC. 3960N First Street, San Jose, CA, 95134). The following reagents were used for Kinetic Turbidimetric LAL assays: Limulus Amebocyte Lysate (Kinetic-QCL TM or PYROGENTTM-5000 Reagent), Control standard Endotoxin (CSE), LAL Reconstitution Buffer (required for the PYROGENT TM-5000 Kinetic Turbidimetric LAL assay), LAL reagent water (LRW, # W50-640, W50-100, W50-500). Endotoxin levels were measured in Group 2.

### Assessments PV loop derived and perfusion parameters

**Figure 2:**
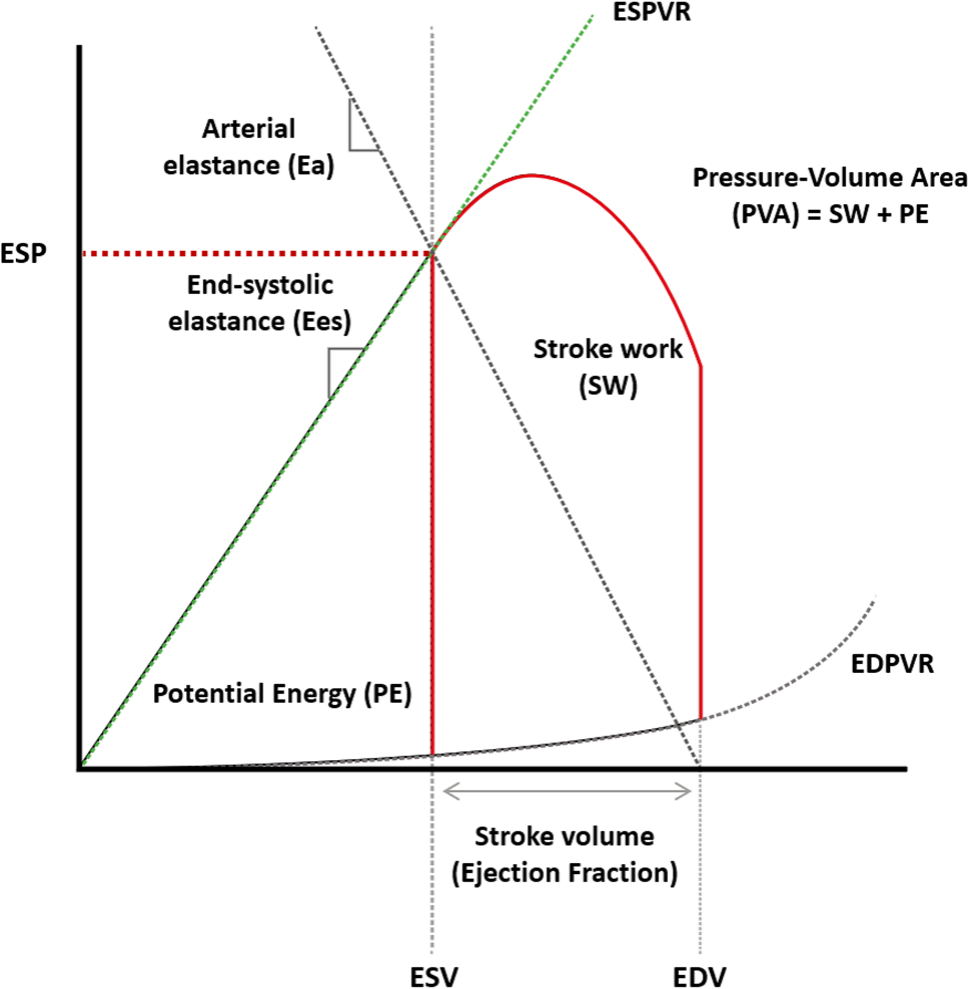
Schematic view of a pressure–volume loop. *EDPVR* end-diastolic pressure–volume relationship, *ESPVR* end-systolic pressure–volume relationship, *ESP* end-systolic pressure, *PVA* pressure–volume area, *EDV* end-diastolic volume, *ESV* end-systolic volume.

The PV loop was recorded for 2 h in all groups. The cardiac parameters were categorized as basic and advanced parameters. Basic parameters include end-diastolic volume (EDV), end-systolic volume (ESV), end-diastolic pressure (EDP), and end-systolic pressure (ESP). The advanced parameters include ejection fraction (EF), end-systolic elastance (Ees), diastolic time constant (Tau) index, arterial elastance (Ea), pressure–volume area (PVA), stroke work (SW), and potential energy (PE). (Fig. 2) To measure changes in contractility, EF and Ees were used. Tau index was used to measure diastolic function; Ea for vascular resistance.

The Ees, the slope of the end-systolic pressure–volume relationship (ESPVR), measures left ventricular contractility^17^. The Tau index, the time constant for isovolumic relaxation, is often used to measure diastolic cardiac function^18^. The Ea calculates the sum of all the extracardiac forces that oppose ventricular ejection or arterial load and is the integrative measure of the arterial system properties^19^. The interaction between the cardiac contractility and the arterial system can be analyzed as the ratio of Ea to Ees which is VAC. Myocardial oxygen consumption can be surmised by the pressure–volume area (PVA)^20^.

As described in Table 1, we set the variables correlated to these PV loop derived parameters as basic and advanced parameters. ECMO flow and lactate levels were categorized as perfusion related parameters.

**Table 1:** Pressure–volume loop derived parameters and perfusion parameters.

	Pre and post variables
**PV loop-derived basic parameters**	End-diastolic pressure (EDP) End-systolic pressure (ESP) End-diastolic volume (EDV) End-systolic volume (ESV)
**PV loop-derived advanced parameters**	
Inotropy/contractility	Ejection fraction (EF) End-systolic elastance (Ees)
Lusitropy/relaxation	Tau index (isovolumetric relaxation time)
Afterload	Arterial elastance (Ea)
Coupling	Ventricular arterial coupling (VAC, ratio of Ea to Ees)
Myocardial oxygen consumption	Pressure volume area (PVA) Potential energy (PE) Stroke work (SW)
**Perfusion related parameters**	ECMO flow Lactate

To measure the myocardial oxygen consumption, we used the variables of PVA, PE and SW. To measure the perfusion, we used variables of ECMO flow and lactate.

### Statistical analysis

Continuous variables are expressed as the median and interquartile range (IQR). These were compared, in each group, before and after the experiment by paired T-test and these variables are expressed as the mean difference (MD) ± standard deviation (SD), 95% confidence intervals (CI). Multivariate logistic regression analysis was performed to test the association between PVA/lactate variables and other cardiac parameters. The multivariate regression model included variables with p < 0.20 in univariate comparisons. We used a stepwise approach, sequentially eliminating variables with a threshold of p > 0.10 to build a final adjusted regression model. A p-value < 0.05 was considered to indicate statistical significance in all analyses. We analyzed the data using R (Version 3.6.3, R Foundation for Statistical Computing, Vienna, Austria) and Rex software (Version 3.6.3, RexSoft Inc., Seoul, South Korea).

## Results

Eighty rats underwent the experiments. Thirty-four rats were included in the analyses: Group 1 (n = 20), Group 2 (n = 14), Group 3 (n = 20) and Group 4 (n = 20). Six rats in Group 2 were excluded due to death before the protocol could be completed and low endotoxin levels less than 5 EU/mL.

### The pre and post PV loops in each group

**Figure 3:**
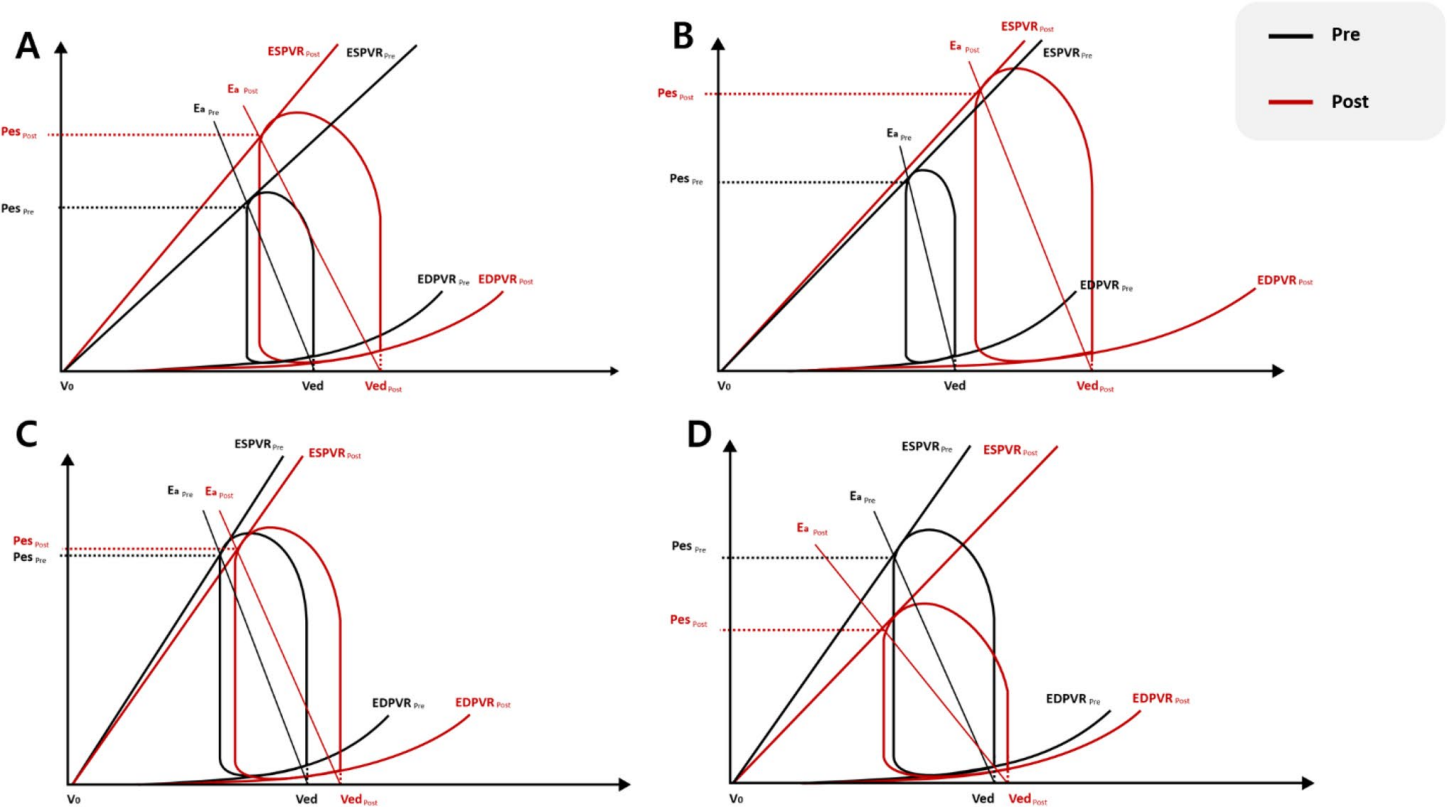
Pressure–volume (PV) loop in each group. The black line represents the baseline PV loop (pre), and the red line represents the PV loop at the end of the experiment. (post). (**A**) PV loop in Group 1. While EDV, ESP, SV, SW, Ees (slope of ESPVR), and PVA increased significantly, the EDP (each point of EDPVR) showed a significant decline after 2 h of ECMO. (**B)** PV loop in Group 2. A significant increase in EDV, ESV, ESP SW and PVA was seen after 2 h of ECMO. (**C**) PV loop in Group 3 (healthy control group). No significant changes in PV derived parameters. (**D**) PV loop in Group 4 (septic control group). EDP, Ea, PVA decreased significantly.

As described in Fig. 3, in group 1 (rats on ECMO), EDV, ESP, SV, SW, Ees (slope of ESPVR), and PVA increased significantly (all P values < 0.05) and the EDP (each point of EDPVR) showed a significant decline (P < 0.001) after 2 h of ECMO; in Group 2, a significant increase in EDV, ESV, ESP SW, and PVA were seen after 2 h of ECMO (all P values < 0.05); in Group 4 (septic rats), EF and SV increased significantly (P = 0.006 and P = 0.003) whereas EDP, Ea, PVA decreased significantly (all P values < 0.05);

### Comparison in mean difference of cardiovascular parameters

As described in Table 2, in Group 1 cardiac parameters changed: the mean difference of ESP (24.0 ± 28.3, 95% CI 10.8 to 37.3, P = 0.001), EDV (50.9 ± 55.0, 95% CI 25.1 to 76.6, P < 0.001), PVA (10.3 ± 11.0, 95% CI 5.1 to 15.4, P < 0.001), PE (5.6 ± 6.8, 95% CI 2.4 to 8.8, P = 0.001), and SW (4.7 ± 5.6, 95% CI 2.1 to 7.4, P = 0.001) increased significantly, while the mean difference of EDP (–2.4 ± 1.8, 95% CI −3.2 to −1.5, P < 0.001) and Tau index (−2.1 ± 3.0, 95% CI −3.5 to −0.6, P = 0.006) significantly declined after 2 h on ECMO. In Group 2, ESP (48.9 ± 22.9, 95% CI 35.7 to 62.2, P < 0.001), EDV (98.9 ± 49.0, 95% CI 70.7 to 127.2, P < 0.001), ESV (66.7 ± 60.5, 95% CI 31.7 to 101.6, P = 0.001), PVA(21.9 ± 11.7, 95% CI 15.1 to 28.6, P < 0.001), PE (16.9 ± 11.3, 95% CI 10.4–23.4, P < 0.001), and SW(4.9 ± 2.9, 95% CI 3.3 to 6.6, P < 0.001 significantly increased after 2 h on ECMO. The ECMO flows were decreased in both groups significantly (P = 0.034 and P = 0.048, respectively) The lactate level increased in both groups significantly. (P < 0.001 and P < 0.001, respectively).

**Table 2:** Within group pre and post comparison of cardiovascular parameters.

	Group 1				Group 2				Group 3				Group 4			
	Pre	Post	Mean difference	P	Pre	Post	Mean difference	P	Pre	Post	Mean difference	P	Pre	Post	Mean difference	P
**Basic parameters**																
EDP (mmHg)	9.7 ± 1.8	7.37 ± 2.5	−2.4	< 0.01	8.9 ± 2.2	8.7 ± 3.3	−0.3	0.61	10.8 ± 2.9	9.6 ± 3.4	−1.1	0.03	8.6 ± 2.9	7.44 ± 3.73	−1.17	0.04
ESP (mmHg)	80.5 ± 13.7	104.6 ± 28.4	24.0	< 0.01	90.0 ± 28.1	139.0 ± 16.5	48.9	< 0.01	110.9 ± 29,8	114.3 ± 18.9	3.36	0.42	104.9 ± 24.9	93.07 ± 33.8	−11.9	0.26
EDV (ml)	237.3 ± 31.1	288.2 ± 45.5	50.9	< 0.01	214.2 ± 31.6	313.2 ± 59.7	98.9	< 0.01	249.2 ± 53.3	266.2 ± 42.24	16.9	0.09	261.5 ± 25.5	277.4 ± 48.8	15.9	0.18
ESV (ml)	181.2 ± 32.4	199.0 ± 53.8	17.8	0.14	168.7 ± 23.4	235.4 ± 60.2	66.7	< 0.01	165.4 ± 46.2	180.8 ± 46.9	15.4	0.13	184.7 ± 43.2	176.2 ± 66.7	−8.4	0.49
***Advanced parameters***																
**Inotropy (contractility)**																
EF%	31.8 ± 7.17	38.2 ± 17.5	6.5	0.08	28.3 ± 7.2	31.3 ± 13.4	3.0	0.40	42.5 ± 7.5	42.2 ± 13.5	−0.33	0.90	37.2 ± 14.2	47.4 ± 20.4	10.1	0.01
Ees	0.5 ± 0.1	0.6 ± 0.3	0.2	0.06	0.5 ± 0.2	0.6 ± 0.2	0.1	0.02	0.7 ± 0.24	0.69 ± 0.27	−0.0165	0.74	0.62 ± 0.29	0.573 ± 0.23	−0.051	0.46
**Lusitropy (relaxation)**																
Tau	14.3 ± 1.7	12.2 ± 2.6	−2.05	< 0.01	13.8 ± 4.2	14.8 ± 4.0	0.9	0.38	13.03 ± 2	9.5 ± 2.5	−3.4	0.00	12.46 ± 3.7	8.5 ± 3.2	−3.9	< 0.01
Afterload																
Ea	1.2 ± 0.5	1.1 ± 0.4	−0.1	0.47	1.7 ± 0.8	1.8 ± 1.1	0.1	0.73	1.2 ± 0.5	1.13 ± 0.37	−0.044	0.68	1.2 ± 0.37	0.89 ± 0.5	−0.31	0.02
**Coupling**																
VAC	2.6 ± 0.9	2.5 ± 1.7	−0.1	0.65	3.1 ± 1.3	2.9 ± 1.5	−0.1	0.77	1.75 ± 0.64	1.92 ± 0.96	0.168	0.27	2.35 ± 1.3	1.91 ± 1.4	−0.441	0.04
**Myocardial oxygen consumption**																
PVA (mmHg ml)	19.2 ± 3.7	29.4 ± 9.7	10.3	< 0.01	19.5 ± .3	41.5 ± 9.4	21.9	< 0.01	26.3 ± 6.3	30.3 ± 6.08	4	0.01	26.6 ± 6.01	29.9 ± 14.5	3.26	0.38
PE (mmHg ml)	14.6 ± 3.6	20.2 ± 6.5	5.5	< 0.01	15.4 ± 5.9	32.4 ± 8.1	16.9	< 0.01	17.9 ± 4.9	20.2 ± 4.8	2.31	0.11	18.7 ± 4.43	17.1 ± 10.1	−1.6	0.55
SW (mmHg ml)	4.5 ± 1.3	9.3 ± 5.4	4.7	< 0.01	4.1 ± 1.8	9.1 ± 4.2	4.9	< 0.01	8.37 ± 2.7	10.06 ± 4.3	1.69	0.04	7.86 ± 3.9	9.7 ± 6.1	1.8	0.18
**Perfusion parameters**																
ECMO flow (ml/min)	36.9 ± 3.8	35.7 ± 4.6	−1.2	0.03	35.9 ± 5.3	33.2 ± 6.2	−2.6	0.04								
Lactate (mg/dl	1.2 ± 0.4	5.4 ± 1.2	4.2	< 0.01	3.1 ± 1.1	11.5 ± 3.6	8.4	< 0.01	1.39 ± 0.42	2,46 ± 0.73	1.07	0.79	3.3 ± 1.27	6.3 ± 2.82	3	0.01

*Ea* arterial elastance, *EDV* end-diastolic volume, *Ees* end-systolic elastance, *EF* ejection fraction, *ESV* end-systolic volume, *PE* potential energy, *PVA* pressure–volume area, *SW* stroke work.

### Comparison of outcome parameters between ECMO groups

WE defined the difference ratio as following: variable (post) − variable (pre)/variable (pre). As described in Table 3, the PVA changed statistically in Group 2 (1.4 ± 0.9) compared to Group 1 (0.6 ± 0.6, P < 0.01) as the PE changed statistically in Group 2 (21.4 ± 1.1) than in Group 1 (10.4 ± 0.6, P < 0.01).

**Table 3:** Between groups pre-post comparison of cardiovascular parameters.

	Group1 (N = 20)	Group2 (N = 14)	P value
**Myocardial oxygen consumption**			
PVA (mmHg ml)	0.6** ± **0.6	1.4** ± **0.9	< 0.01
PE (mmHg ml)	0.4** ± **0.6	1.4** ± **1.1	< 0.01
SW (mmHg ml)	1.2** ± **1.3	1.3** ± **0.9	0.88
**Perfusion parameters**			
ECMO flow (ml/min)	−0.03** ± **0.07	−0.07** ± **0.12	0.23
Lactate(mg/dl)	4.0** ± **2.2	3.0** ± **1.8	0.17

*PE* potential energy, *PVA* pressure–volume area, *SW* stroke work.

### Regression between myocardial oxygen consumption and other parameters in septic rats on ECMO

As described in Table 4, univariate linear regression analyses showed that Ees, Tau, and Ea were significantly correlated with PVA (P < 0.01, P < 0.01, and P = 0.01, respectively). However, only the Tau index was significantly correlated with PVA after multivariate analyses (β = 0.12, SE = 0.05, P = 0.02).

**Table 4:** Regression for PVA to other PV loop-derived advanced parameters and perfusion parameters in the 14 septic rats on ECMO.

	Univariate			Multivariate		
	β	95% CI	P	β	SE	P
***Advanced parameters***						
**Inotropy (contractility)**						
EF (%)	0.05	0.03	0.16			
Ees	−4.49	1.19	< 0.01	−1.58	1.26	0.19
**Lusitropy (relaxation)**						
Tau	0.19	0.04	< 0.01	0.12	0.05	0.02
**Afterload**						
Ea	−0.73	0.25	0.01	−0.25	0.21	0.18
**Coupling**						
VAC	−0.25	0.20	0.23			
**Perfusion parameters**						
ECMO flow(ml/min)	0.00	0.05	0.99			
Lactate(mg/dl)	0.13	0.26	0.60			

*Ea* arterial elastance, *EDV* end-diastolic volume, *Ees* end-systolic elastance, *EF* ejection fraction, *ESV* end-systolic volume, *PE* potential energy, *PVA* pressure–volume area, *SW* stroke work.

## Discussion

IN this in vivo study, we compared two groups of rats supported on ECMO for 2 h, one with induced sepsis and one control group. We investigated the changes of parameters derived from PV loops and perfusion parameters such as ECMO flow and lactate level. The PVA, a surrogate of myocardial oxygen consumption, rose significantly higher in the septic rats than in the healthy rats on ECMO. We analyzed the relationship between myocardial oxygen consumption after 2 h of ECMO run and other baseline parameters in septic rats on ECMO, resulting in the Tau index (relaxation parameter) significantly correlated with PVA.

### Pre and post-comparison of cardiovascular parameters within the group

Each experimental group showed characteristic changes in cardiovascular parameters after 2 h of PV loop monitoring. In group 1, the difference in PVA and Tau was induced as an adaptive response to the ECMO itself. In group 2, where myocardial inflammation was induced by endotoxemia, there was no change in myocardial contraction and relaxation, but secondary adaptation occurred through ventricular dilatation.

### Comparison between septic rats vs. healthy rats on ECMO

AS the baselines of both groups were different, we used changing ratio, defined as variable (post)-variable (pre)/variable (pre). The changing ratio of PVA and PE was significantly higher in Group 2, which can be interpreted as myocardial oxygen consumption being higher in septic rats on ECMO than in healthy rats on ECMO. As PE is also a component of PVA, we decided to investigate further the relationship between PVA and other parameters in septic rats on ECMO.

### Relationship between myocardial oxygen consumption (PVA) and other parameters in septic rats on ECMO

To elucidate the relationship between myocardial oxygen consumption and other parameters in septic rats on ECMO, PVA was used as a surrogate for myocardial oxygen consumption. In multivariate analyses, the Tau index was significantly correlated with PVA in septic rats on ECMO.

It is known that ECMO itself exerts an additional load on the left ventricle^21^. PVA is the cardiac parameter that represents the total mechanical energy generated by ventricular contraction. It is closely correlated to left ventricular oxygen consumption regardless of the loading conditions of the heart^7,22^. PVA significantly increased in response to ECMO application in both groups. These results corroborate with evidence from human studies that ECMO can increase myocardial oxygen consumption, presumably by increasing LV wall tension^20^. While the sum of SW and PE constructs PVA, SW and PE were increased in both groups. However, when we compared SW, PE, and PVA between the groups, the changes in PVA and PE were more dominant in group 2. The increase in PE will increase the myocardial oxygen consumption, which will require more oxygen for the heart to recover^17^. In addition, the myocardial oxygen consumption (PVA) was most significantly affected in septic rats on ECMO. This is the result of the two groups reacting differently to ECMO support.

Of note, diastolic dysfunction is commonly seen in patients with sepsis^23^. In this study, we obtained the Tau index referring to diastolic function; it decreased in healthy rats after 2 h on ECMO; however, in septic rats, the Tau index tended to increase, although we could not find statistical significance. The relationship between PVA and other PV loop-derived advanced parameters showed that only the Tau index was significantly associated with PVA. This result can be interpreted as diastolic dysfunction associated with increased myocardial oxygen consumption, which may be associated with higher mortality in patients with septic shock on ECMO.

Our study has some limitations. While our experiment aimed to mimic ECMO support in humans, the ECMO flow was relatively low, and the rats were continuously infused with albumin to maintain hemodynamic parameters and in septic rats with diastolic dysfunction with increased fluid administration might have led to increased mortality^23^.

In conclusion, myocardial oxygen consumption was higher in septic shock with ECMO than in controls. In addition, diastolic dysfunction was associated with increased myocardial oxygen consumption in septic rats on ECMO.

## Data Availability

The datasets used and/or analyzed during the current study available from the corresponding author on reasonable request.

## Abbreviations

Ea: Arterial elastance
ECMO: Extracorporeal membrane oxygenation
EDP: End-diastolic pressure
EDPVR: End-diastolic pressure–volume relationship
EDV: End-diastolic volume
Ees: End-systolic elastance
EF: Ejection fraction
ESP: End-systolic pressure
ESPVR: End-systolic pressure–volume relationship
ESV: End-systolic volume
LPS: Lipopolysaccharide
PE: Potential energy
PVA: Pressure–volume area
SV: Stroke volume
SW: Stroke work
Tau: Diastolic time constant
VA: Venoarterial
VAC: Ventricular arterial coupling
