# Author Correction: Combined effects of sepsis and extracorporeal membrane oxygenation on left ventricular performance in a murine model

**DOI:** 10.1038/s41598-023-28375-9

**Published:** 2023-01-19

**Authors:** Mukhammad Kayumov, Dowan Kim, Sainath Raman, Graeme MacLaren, In Seok Jeong, Hwa Jin Cho

**Affiliations:** 1 Chonnam National University Graduate School, Gwangju, Republic of Korea; 2 Department of Thoracic and Cardiovascular Surgery, Chonnam National University Hospital and Medical School, 42 Jebong-ro Donggu, Gwangju, Republic of Korea; 3 Centre for Children’s Health Research, The University of Queensland, Brisbane, QLD Australia; 4 Cardiothoracic ICU, National University Hospital, Singapore, Singapore; 5 Department of Pediatrics, Chonnam National University Children’s Hospital and Medical School, 42 Jebong-ro Donggu, Gwangju, Republic of Korea

Correction to: *Scientific Reports* https://doi.org/10.1038/s41598-022-26145-7, published online 23 December 2022

The Funding section in the original version of this Article was omitted.

The Funding section now reads: “This study was supported by grants (BCRI20018, BCRI22022 and BCRI 22025) from the Chonnam National University Hospital Biomedical Research Institute to DWK, ISJ and HJC, by Chonnam National University (2021-2443 and 2022-3856-01) to DWK and HJC. This study was also supported by grants from National Research Foundation (NRF-2021R1I1A3047390, NRF-2019R1D1A3A03103899, NRF-2020R1F1A1073921) to DWK, ISJ and HJC.”

The original Article has been corrected.

